# Serum metabolites in relation to kidney function in non-transplant and kidney transplant settings using gas chromatography-mass spectrometry

**DOI:** 10.1186/s12882-026-05000-1

**Published:** 2026-04-29

**Authors:** Yamei Li, Xingxin Gong, Yangjuan Bai, Lin Yan, Yunfei An, Hanjing Liu, Hua Zhang, Xinhua Dai

**Affiliations:** 1 Department of Laboratory Medicine, West China Hospital, Sichuan University, Chengdu, Sichuan province China; 2 Clinical Laboratory Medicine Research Center of West China Hospital, Chengdu, Sichuan province China; 3 Sichuan Clinical Research Center for Laboratory Medicine, Chengdu, Sichuan province China; 4 Department of Pathology, General Hospital of Western Theater Command, Chengdu, Sichuan province China

**Keywords:** Metabolomics, Chronic kidney disease, Kidney transplantation, Gas chromatography-mass spectrometry

## Abstract

**Background:**

Metabolic disorders are highly prevalent in patients with native and transplanted chronic kidney disease (CKD). However, little is known about the metabolic similarities and differences associated with declining kidney function between non-transplant patients and kidney transplant recipients (KTRs).

**Methods:**

This cross-sectional study employed gas chromatography-mass spectrometry to compare serum metabolomic profiles among native CKD subgroups and KTR subgroups, aiming to identify differential metabolites associated with kidney dysfunction in both clinical settings.

**Results:**

11 and 13 metabolites were associated with kidney dysfunction in native CKD patients and KTRs, respectively. Among these, L-tryptophan, D-lyxose, xylitol, erythritol, 3,4-dihydroxybutanoic acid and 2,4-dihydroxybutanoic acid were selected as common differential metabolites in both cohorts. Pathway analysis revealed that the pentose and glucuronate interconversions pathway was significantly affected in native CKD patients, whereas phenylalanine, tyrosine, and tryptophan biosynthesis pathway and tyrosine metabolism pathway were the most affected pathways in KTRs. Further comparisons between native CKD and KTRs who were at the same kidney dysfunction stages demonstrated that KTRs showed significantly higher levels of malic acid, but lower levels of D-allose compared to native CKD patients.

**Conclusions:**

Our study not only identified the 6 metabolites associated with kidney function in both non-transplant patients and KTRs, but also determined 5 and 7 metabolites that were specifically associated with kidney dysfunction in native CKD patients and KTRs, respectively. These data suggest that the metabolic process may be influenced by the unique characteristics of kidney transplantation, such as allogeneic immunity and immunosuppressive drugs, in KTRs.

**Supplementary Information:**

The online version contains supplementary material available at 10.1186/s12882-026-05000-1.

## Introduction

Chronic kidney disease (CKD) is a major public health problem affecting 11–13% of the population globally [1]. The irreversible progression of CKD often leads to end-stage kidney disease (ESKD), which requires renal replacement therapy, such as dialysis and kidney transplantation (KT), to extend patient survival and improve quality of life. As the most effective treatment for ESKD, KT could successfully and immediately restore the estimated glomerular filtration rate (eGFR) and endocrine function of the patients, thereby improving their life expectancy. However, renal allografts do not last forever due to the occurrence of rejection, infection, and immunosuppressive drug-related side effects. Approximately 40% of the kidney transplant recipients (KTRs) develop chronic allograft dysfunction (CAD) 10 years after KT [2]. In 2005, KDIGO defined KTRs as a unique subgroup of patients with CKD considering the presence of a single functional allo-kidney, lifelong immunosuppressive drug therapy, and long-term pre-existing CKD disease [3]. Currently, serum creatinine (SCR) and eGFR are routinely used in clinical practice as indicators of kidney function; however, disturbances in their levels only occur when the kidney filtration capacity is significantly lost, and therapeutic interventions may be ineffective at this time [4]. Revealing the common pathophysiological nature underlying kidney dysfunction in non‑transplant CKD patients and KTRs may provide insights into shared pathways and identified candidate molecules that could be further investigated as potential universal biomarkers.

Metabolites are downstream products of genes and proteins that reflect the physiological state and perform specific functions. As the second-highest metabolic rate organ, the kidney when damaged significantly alters the concentration and composition of metabolites, which may aid in the discovery of candidate biomarkers and potential therapeutic targets. A series of studies revealed that metabolic disorders were highly prevalent in both native and transplanted CKD patients [5, 6]. Although KTRs are defined as a subset of CKD and are susceptible to ESKD, the eGFR decline rate is significantly lower in KTRs than in non-transplant CKD patients with similar levels of kidney function [7]. Moreover, alloreactive immune regulation and the pharmacological effects of immunosuppressive drugs endow KTRs with unique characteristics that are distinct from those of native CKD patients. Whether and how these differences influence metabolic profiles in relation to kidney dysfunction remains to be investigated in non-transplant and transplant patients.

Therefore, in the present study, we used gas chromatography-mass spectrometry (GC-MS) to compare the small-molecule metabolites in the serum of patients with native CKD and transplanted CKD, with the aim of identifying and characterizing the specific serum metabolites that are closely related to cross-sectional measures of kidney function in both native and transplanted CKD patients. Meanwhile, similarities and differences between native and transplanted CKD patients with respect to metabolic alterations were also investigated.

## Methods

### Patients and sample collection

This cross-sectional study included two cohorts of study populations from the West China Hospital of Sichuan University from July 2021 to May 2022. As the worsening of renal function is clinically determined by elevated SCR and decreased eGFR, we grouped CKD patients based on eGFR levels in the present study. A total of 196 participants were included, a sample size based on feasibility and data stability considerations for this exploratory metabolomic study. In cohort 1, 87 non-transplant CKD patients were classified into CKD1 (60 ≤ eGFR < 90 ml/min/1.73m^2^), CKD2 (30 ≤ eGFR < 60 ml/min/1.73m^2^), and CKD3 (eGFR < 30 ml/min/1.73m^2^) subgroups. In cohort 2, 86 KTRs with different renal allograft functions were enrolled and were classified into CAD1, CAD2, and CAD3 subgroups based on eGFR levels were between 60 and 90, 30–60, and less than 30 ml/min/1.73m^2^, respectively. 23 healthy volunteers were enrolled as the controls. The inclusion criteria for CKD patients were as follows: (1) age > 18 years old; (2) with varying degrees of renal dysfunction (eGFR < 90 ml/min/1.73m^2^); and (3) without complications of active metabolic diseases other than the underlying cause of CKD. The inclusion criteria for KTRs were as follows: (1) age at transplantation time > 18 years old; (2) received a standard triple regimen (tacrolimus + mycophenolate mofetil + prednisone) as maintenance immunosuppression; and (3) without complications of metabolic diseases. The inclusion criteria for healthy controls (HCs) were as follows: adult physical examiners whose laboratory examination tests (including routine blood tests, biochemical tests, tumor biomarkers, and thyroid function) showed no abnormalities. Serum was selected for the present metabolomic analyses as it is the standard sample type used in our hospital for routine biochemical tests, which enables seamless integration with clinical workflows. Serum samples were collected after centrifugation and stored at -80 °C until the determination of metabolites. This study was approved by the institutional review board of the West China Hospital of Sichuan University (No.2017 − 397) and all participants provided written informed consent prior to enrollment.

### GC-MS analysis and data processing

Detailed information on reagents and solutions, serum sample preparation, data acquisition, and data processing of GC-MS analysis was the same as previously described [8]. Briefly, 100 µL 4℃ rewarmed serum was pretreated with 300 µL cooled acetonitrile for deproteinization. After the supernatant had dried, methylhydroxylamine hydrochloride and BSTFA (containing 1% TMCS) were used for derivatization. The supernatant was loaded on a GC-MS system (TQS9000, Thermo Fisher Scientific, USA) which is equipped with an automatic injector and a TG-5MS capillary column (30 m × 0.25 mm, 0.25 μm, Thermo Fisher Scientific, USA) for further analysis. Quality control (QC) was used to evaluate the reproducibility of the method.

The column oven temperature raised from 60 ℃ to 300 ℃ at a rate of 8 ℃/min and lasted for 7 min. Purified helium (≥ 99.999%) at a constant flow rate of 1.0 mL/ min was used as the carrier gas. Mass spectrometry detection was performed in electron ionization mode at 70 eV to exert a full scan range of 50–600 m/z. Raw data were obtained and processed using Chromeleon 7.3 (Thermo Fisher Scientific, USA) and GC Deconvolution software (Thermo Fisher Scientific, USA), respectively. Peaks were identified when the matching scores were greater than 700 in comparison with data from the National Institute of Standards and Technology (NIST) library 2014. The retention index (RI) of each metabolite was determined based on the working solutions of the RI. Deviations in the RI for all metabolites were calculated by comparing their respective values to those in the database. Additionally, a peak alignment approach was employed to correct the retention times of THE characteristic ions, with a deviation in the RI within 2%. The relative concentrations of metabolites were calculated by dividing the peak area of the metabolite in the sample by that of the corresponding internal standard, which was filtered out with the lowest relative standard deviation (RSD). Metabolites with an RSD of > 30% in the QC samples were removed from the rest of the analysis. All serum samples from HCs, native CKD patients, and KTRs were processed, analyzed, and quantified randomly in a single batch using the identical GC-MS protocol and platform to ensure methodological consistency and comparability across all study groups. The raw metabolomics data generated in this study have been deposited in the National Genomics Data Center (NGDC) OMIX repository under accession code OMIX013657.

### Statistical analyses

Metabolites concentrations were normalized relative to the HC group, with the HC median value set to 1, thereby expressing all data as fold changes for direct comparison across groups. Partial least squares-discriminant analysis (PLS-DA) was applied for dimensionality reduction and to show separation among the groups. Heatmaps were generated to visualize the clustering and the altered metabolites among the groups. To obtain the differential metabolites that were related to native CKD and CAD severity, intersected metabolites were further filtered from group comparison results based on the condition that variable influence on projection (VIP) values > 1.0, fold changes (FC) > 1.2 or < 0.83 and *P* < 0.05. The major metabolic pathways that are involved in common differential metabolites were identified with -Log10 (p) > 1.3 and impact score > 0.1 in KEGG pathway data analysis [9]. To compare metabolic profiles between native CKD and KTRs at equivalent levels of kidney dysfunction, we conducted cross-cohort comparisons (CKD1 vs. CAD1, CKD2 vs. CAD2, CKD 3 vs. CAD3). All the above analyses were completed using MetaboAnalyst 6.0 (https://www.metaboanalyst.ca).

Data are presented as number, mean ± standard deviation, or median (interquartile range) according to the data type. The chi-square test was used to compare categorical variables between groups. The Student’s t-test or Mann-Whitney U test was used to compare continuous variables with normal and skewed distributions, respectively. The Spearman correlation matrix was built with the “corrplot” package in R software (version 4.2.0) to assess the potential confounders (such as age, NLR). However, formal confounder-adjusted effect estimates were not calculated for individual metabolites due to the exploratory nature of this study and the high dimensionality of the metabolic data. Participants with missing data for a specific analysis were excluded from that particular analysis. Statistical analyses were performed using SPSS software (Version 23.0, SPSS Inc., Chicago, IL, USA), and a two-tailed *P* value < 0.05 was considered statistically significant. Graphs were generated using GraphPad Prism (Version 9.0, GraphPad, La Jolla, CA, USA). Venn’s grams were obtained from an online website (https://bioinfogp.cnb.csic.es/tools/venny/index.html).

## Results

### Characteristics of study populations

**Fig. 1:**
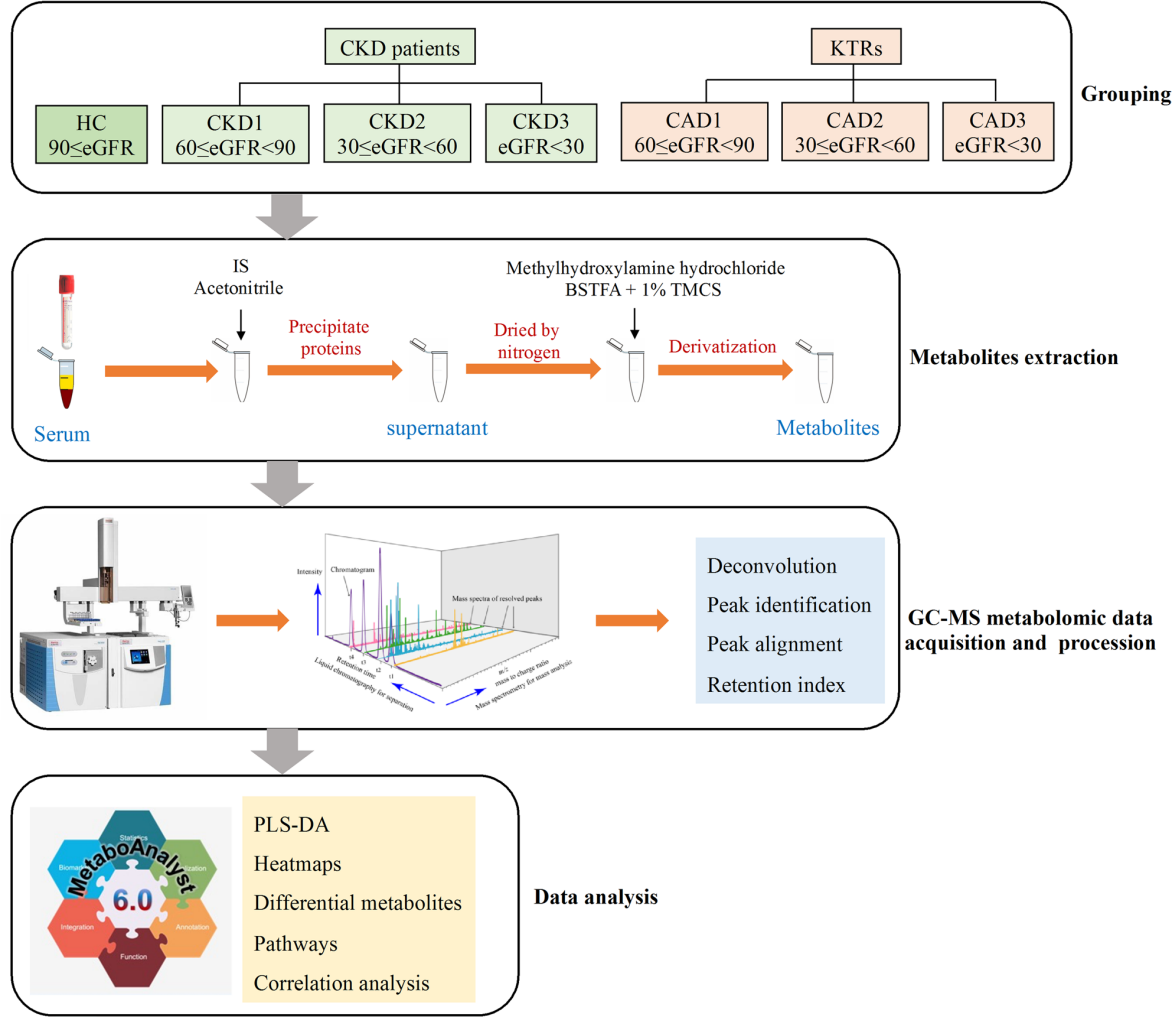
The workflow for data analysis of GC-MS-based metabolomics of native CKD patients and KTRs. Note: CKD, chronic kidney disease; KTRs, kidney transplant recipients; CAD, chronic allograft dysfunction; eGFR, estimated glomerular filtration rate; IS, internal standard; BSTFA, bis(trimethylsilyl)trifluoroacetamide; TMCS, chlorotrimethylsilane. GC-MS, gas chromatography-mass spectrometry; PLS-DA, partial least squares-discriminant analysis

The overall study design is illustrated in Fig. 1. The demographic and baseline clinical data of the participants in the native CKD and KTR groups are summarized in Tables 1 and 2, respectively. The most frequent underlying etiologies among native CKD patients were IgA nephropathy, hypertensive nephropathy, and chronic glomerulonephritis. In contrast, antibody-mediated rejection (ABMR) and recurrent glomerulonephritis were common causes of CAD in KTRs. Consistent with the grouping criteria, the renal function-associated markers (including SCR, eGFR, serum urea, serum cystatin C and urine protein) were significantly different among the 3 CKD subgroups and 3 KTR subgroups and showed the expected alteration trends. As shown in Table 1, patients in the CKD groups were older than HCs, while other parameters such as gender distribution, ALT, and lipid profile are not different among the groups. Although the liver function marker AST was statistically different among the groups, the difference was believed to be of non-clinical significance because all levels were within normal ranges. As shown in Table 2, KTRs in the CAD1 and CAD3 groups showed significantly longer post-transplantation times than those in the CAD2 group, while no difference was observed among groups regarding age, gender, trough concentration of tacrolimus, liver function, and lipid profiles. Additionally, the CAD3 group had an elevated NLR compared to both the CAD1 and CAD2 groups.

**Table 1:** characteristics of native CKD patients and HCs

	HC	CKD1	CKD2	CKD3	*P*
Number	23	27	30	30	/
Age, years	35.4 ± 6.7	40.8 ± 10.1	47.4 ± 13.2	47.5 ± 15.5	0.001
Male, n (%)	21 (91.3%)	16 (59.3%)	21 (70.0%)	22 (73.3%)	0.086
**Underlying etiologies of CKD**					0.060
IgA nephropathy	/	8	4	3	
Hypertensive nephropathy	/	3	12	7	
Chronic glomerulonephritis	/	4	6	4	
Diabetic nephropathy	/	1	2	6	
Others	/	3	0	1	
Unknown	/	8	6	9	
**Kidney function**					
SCR (µmol/l)	79.3 ± 9.1	97.5 ± 14.0	156.3 ± 29.5	561.2 ± 304.3	< 0.0001
eGFR (ml/min/1.73m^2^)	106.5 ± 8.8	75.2 ± 9.1	42.5 ± 7.6	11.9 ± 6.5	< 0.0001
Serum urea, mmol/L	4.5 ± 1.1	6.4 ± 2.3	8.4 ± 2.7	20.9 ± 9.1	< 0.0001
Serum cystatin C, mg/L	0.8 ± 0.1	1.3 ± 0.4	1.9 ± 0.4	4.6 ± 1.9	< 0.0001
Urine protein, n					< 0.0001
0	20	12	5	2	
+/-	2	5	4	1	
+	1	3	4	4	
++	0	6	10	11	
+++	0	0	4	4	
Unavailable	0	1	3	8	
**Liver function**					
ALT, IU/L	22 (17–30)	17 (11–23)	17 (12–21)	15 (8–27)	0.101
AST, IU/L	19 (16–22)	21 (15–25)	20 (16–24)	14 (11–19)	0.034
**Lipid profile**					
Total cholesterol, mmol/L	4.55 (4.22–4.94)	4.20 (3.71–4.84)	4.19 (3.43–5.05)	4.21 (3.39–4.71)	0.229
Triglyceride, mmol/L	1.47 (1.04–1.77)	1.52 (1.25–1.65)	1.48 (1.15–2.28)	1.53 (1.14–2.07)	0.837
HDL-C, mmol/L	1.20 (1.07–1.50)	1.12 (1.03–1.36)	1.07 (0.94–1.32)	0.78 (1.00-1.41)	0.053
LDL-C, mmol/L	2.70 (2.27–3.11)	2.68 (1.90–3.01)	2.56 (1.98–3.22)	2.15 (1.93–2.73)	0.184
**Others**					
NLR	1.72 (1.51–2.55)	1.93 (1.59–2.99)	2.60 (2.06–3.28)	2.71 (1.88–3.44)	0.054

Abbreviations: CKD, chronic kidney disease; HC, health control; SCR, serum creatinine; eGFR, estimated glomerular filtration rate; ALT, alanine aminotransferase; AST, aspartate transaminase; HDL-C, high-density lipoprotein cholesterol; LDL-C, low-density lipoprotein cholesterol; NLR, neutrophil-to-lymphocyte ratio

**Table 2:** Characteristics of KTRs

	CAD1	CAD2	CAD3	*P*
Number	30	30	26	/
Age, years	42.1 ± 11.2	39.1 ± 8.6	37.4 ± 10.0	0.208
Male, n (%)	22 (73.3%)	22 (73.3%)	17 (65.4%)	0.757
Time after KT, years	5.53 (4.08–7.18)	3.55 (1.82–5.44)	5.46 (3.22–8.59)	0.015
Trough concentration of tacrolimus (ng/ml)	5.09 (4.29–6.57)	5.89 (4.57–6.50)	4.58 (3.34–6.64)	0.133
**Underlying etiologies of CAD**				0.045
ABMR	/	6	12	
Recurrent Glomerulonephritis	/	8	1	
Others	/	2	3	
Unknown	/	14	10	
**Kidney function**				
SCR (µmol/l)	99.2 ± 17.5	170.3 ± 26.0	292.9 ± 82.5	< 0.0001
eGFR (ml/min/1.73m^2^)	76.4 ± 10.0	40.7 ± 6.8	21.7 ± 4.8	< 0.0001
Serum urea, mmol/L	6.1 ± 1.5	10.2 ± 3.2	17.9 ± 7.1	< 0.0001
Serum cystatin C, mg/L	1.2 ± 0.2	2.1 ± 0.6	3.5 ± 1.0	< 0.0001
Urine protein, n				0.013
0	17	13	4	
+/-	6	4	4	
+	5	6	5	
++	2	4	11	
+++	0	3	2	
**Liver function**				
ALT, IU/L	13 (10–21)	13 (10–16)	14 (10–22)	0.421
AST, IU/L	17 (15–21)	16 (14–19)	19 (12–23)	0.522
**Lipid profile**				
Total cholesterol, mmol/L	5.03 (4.48–5.50)	5.25 (4.51–5.77)	4.96 (3.52–5.39)	0.168
Triglyceride, mmol/L	1.33 (0.98–2.04)	1.87 (1.35–2.42)	1.52 (1.05–2.27)	0.121
HDL-C, mmol/L	1.36 (1.08–1.53)	1.28 (1.10–1.63)	1.14 (1.00-1.73)	0.869
LDL-C, mmol/L	3.02 (2.33–3.38)	2.97 (2.31–3.60)	2.60 (1.80–3.21)	0.149
**Others**				
NLR	2.11 (1.88–2.53)	2.47 (1.72–3.74)	4.25 (2.68–5.66)	0.001

Abbreviations: KTRs, kidney transplant recipients; CAD, chronic allograft dysfunction; ABMR, antibody-mediated rejection; SCR, serum creatinine; eGFR, estimated glomerular filtration rate; ALT, alanine aminotransferase; AST, aspartate transaminase; HDL-C, high-density lipoprotein cholesterol; LDL-C, low-density lipoprotein cholesterol; NLR, neutrophil-to-lymphocyte ratio

### Identification of differential metabolites associated with kidney dysfunction in native CKD patients and KTRs

**Fig. 2:**
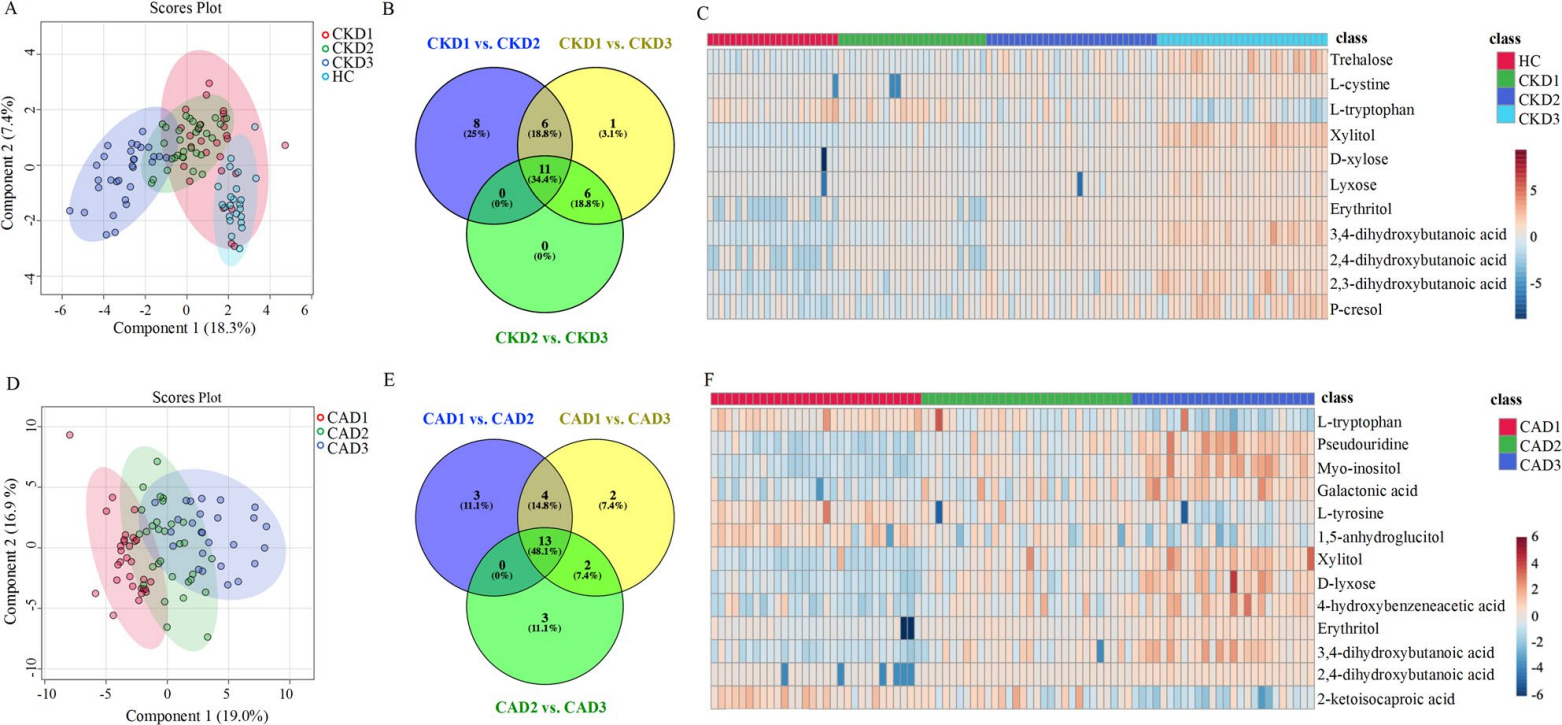
Identification of the differential metabolites within CKD subgroups and KTR subgroups. (**A**) PLS-DA score plot of serum metabolic profiles among CKD subgroups and HC group. (**B**) Venn diagram depicting the number of commonly altered metabolites in CKD patients with different renal functions: CKD1 vs. CKD2 (blue), CKD1 vs. CKD3 (yellow), CKD2 vs. CKD3 (green). (**C**) The heatmap of common differential metabolites among CKD subgroups; (**D**) PLS-DA score plot of serum metabolic profiles among KTR subgroups. (**E**) Venn diagram depicting the number of commonly altered metabolites in KTRs with different allograft functions: CAD1 vs. CAD2 (blue), CAD1 vs. CAD3 (yellow), CAD2 vs. CAD3 (green); (**F**) The heatmap of common differential metabolites among KTR subgroups. Note: CKD, chronic kidney disease; KTR, kidney transplant recipient; PLS-DA, partial least squares-discriminant analysis; HC, healthy control

**Fig. 3:**
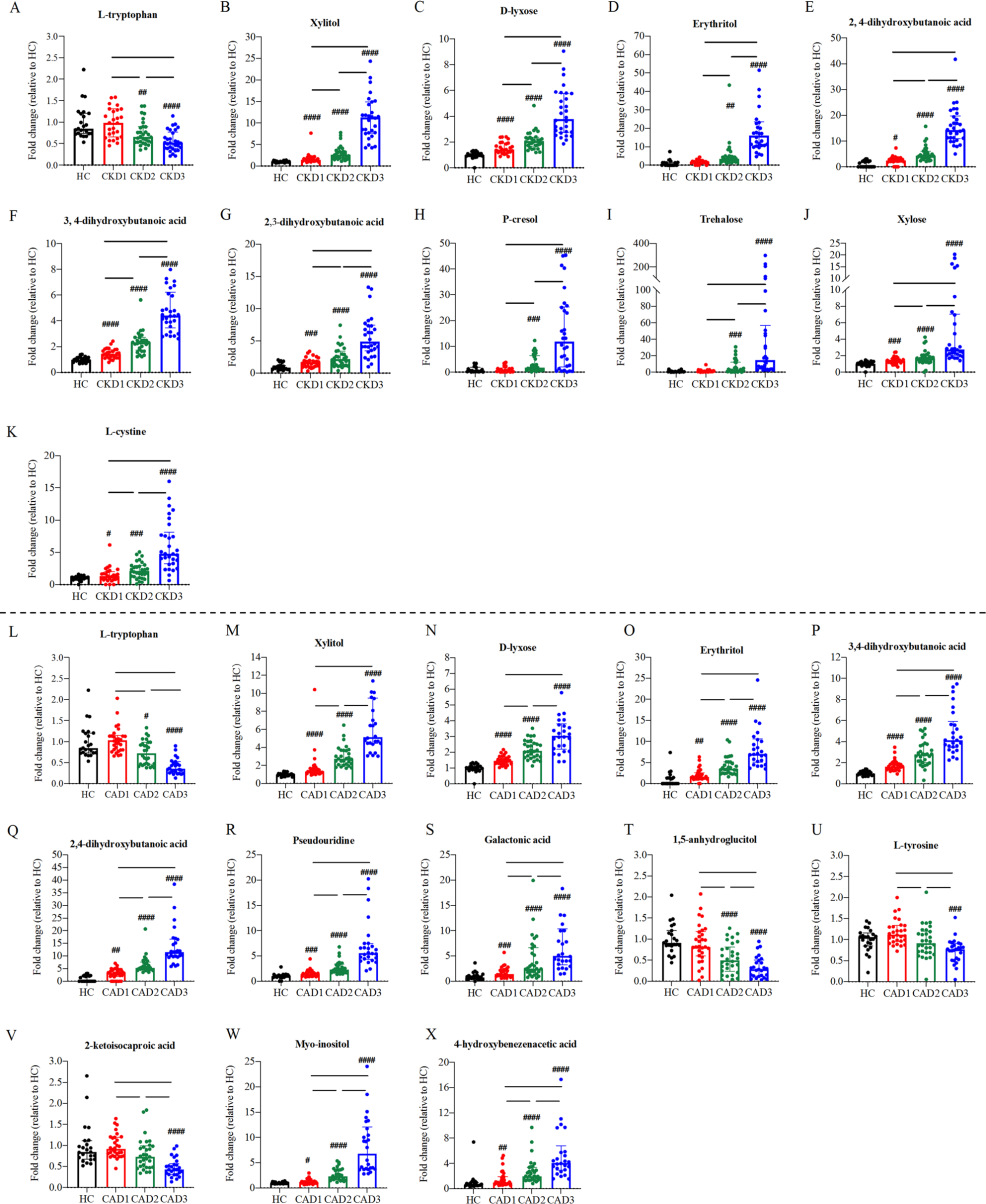
The relative intensities of individual differential metabolites among the groups. **A**-**K**. Differences in abundance of 11 commonly altered metabolites among the HC and 3 CKD subgroups (upper panel for CKDs). **L**-**W**. Differences in abundance of 13 commonly altered metabolites among the 3 KTR groups (lower panel for KTRs). Note: #Compared with HC group, # P < 0.05; ##P < 0.01; ###P < 0.001; ####P < 0.0001; *P < 0.05; **P < 0.01; ***P < 0.001; ****P < 0.0001. CKD, chronic kidney disease; KTR, kidney transplant recipient; CAD, chronic allograft dysfunction; HC, healthy control

PLS-DA plots were established to visualize the overall metabolic profiling differences and similarities. As shown in Fig. 2A and D, there were clear separations of CK(A)D subgroups on the PLS-DA plots of serum metabolites, with the HC, CK(A)D1, CK(A)D2, and CK(A)D3 separated linearly in numeric orders. To select the key metabolites related to different levels of kidney function that affected the clustering tendency within the CKD and CAD subgroups, pairwise comparisons were first conducted within 3 CKD and 3 KTR subgroups. The metabolites were initially screened out with selection criteria of VIP > 1.0, the *P* < 0.05, and the fold change (FC) ≥ 1.2 or ≤ 0.83 by comparing the metabolites between CK(A)D1 and CK(A)D2 groups, CK(A)D2 and CK(A)D3 groups, and CK(A)D1 and CK(A)D3 groups. The preliminarily selected metabolites were intersected as the common differential metabolites. As a result, 11 and 13 common differential metabolites were identified by comparing the 3 CKD subgroups and 3 KTR subgroups, respectively. The heatmap visualization of common differential metabolites portrayed the apparent discrimination of patients within different groups (Fig. 2C and F). Further comparison of the relative intensities of individual differential metabolites among groups showed that the level of 1 metabolite (L-tryptophan) decreased, and levels of 10 metabolites (lyxose, 2,4-dihydroxybutanoic acid, 3,4-dihydroxybutanoic acid, erythritol, xylitol, p-cresol, trehalose, 2,3-dihydroxybutanoic acid, xylose and L-cystine) increased with more severe renal dysfunction in CKD groups (Fig. 3A-K), while levels of 4 metabolites (L-tryptophan, 1,5-anhydroglucitol, L-tyrosine and 2-ketoisocaproic acid) decreased, and levels of 9 metabolites (lyxose, 2,4-dihydroxybutanoic acid, 3,4-dihydroxybutanoic acid, erythritol, xylitol, pseudouridine, galactonic acid, myo-inositol and 4-hydroxybenzeneacetic acid) increased with the allograft dysfunction severity in KTR groups (Fig. 3L-W).

### Metabolic pathways associated with kidney dysfunction in native CKD patients and KTRs

**Fig. 4:**
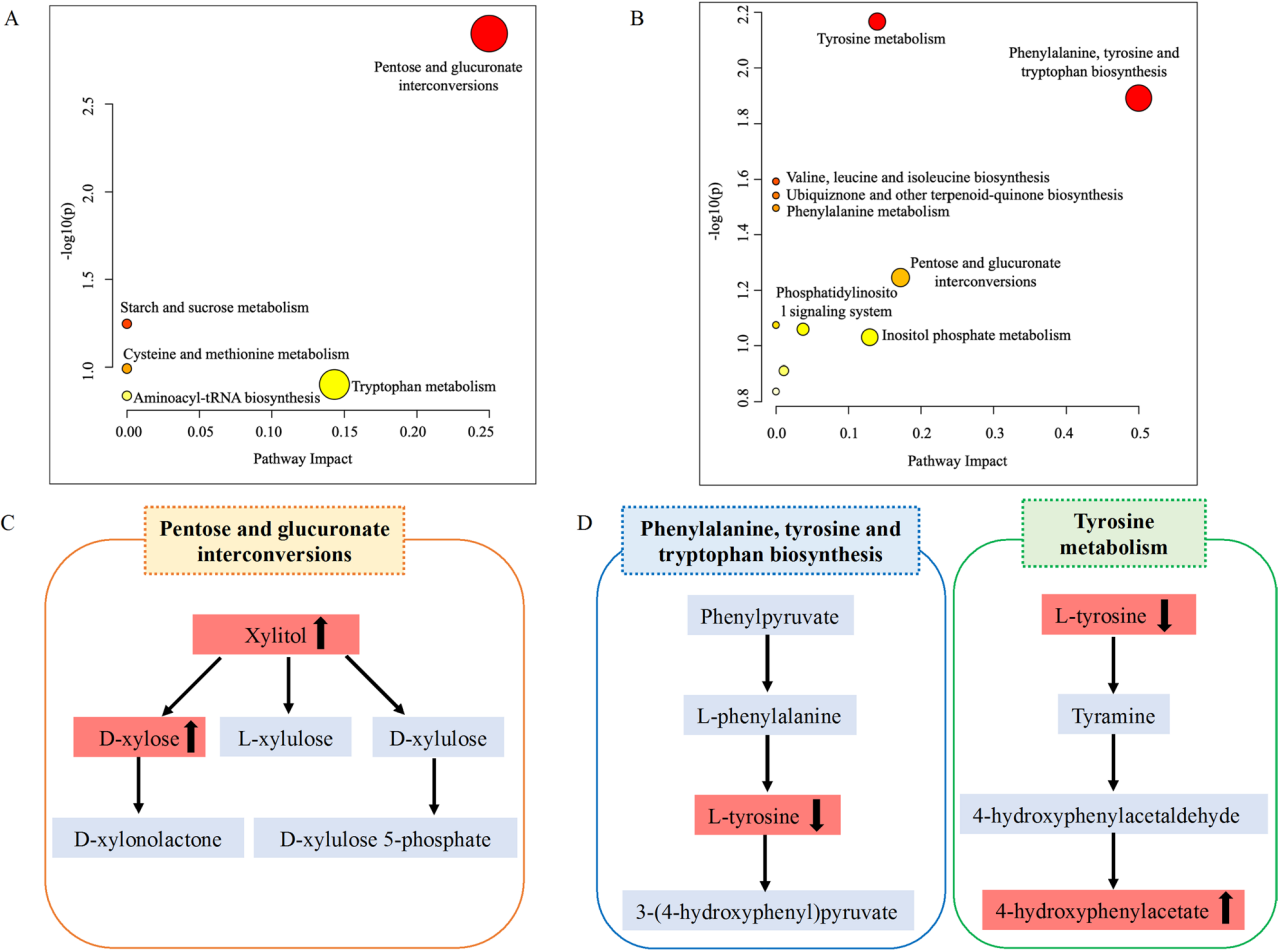
Pathway analysis of differential metabolites found in serum samples among native CKD and KTR subgroups. **A**-**B**. Metabolic topological analysis diagrams of differential metabolites based on the Kyoto Encyclopedia of Genes and Genomes (KEGG) online database using MetaboAnalyst 6.0 in native CKD (**A**) and KTR (**B**) subgroups. **C**-**D**. The interactive networks of the pathways identified for kidney disease progression in the native CKD (**C**) and KTR (**D**) subgroups. Note: CKD, chronic kidney disease; KTR, kidney transplant recipient

The metabolic pathway analysis was further carried out based on the KEGG database through MetaboAnalyst 6.0. The results demonstrated that pentose and glucuronate interconversions was identified as the key pathway that was closely associated with the severity of native CKD (Fig. 4A). Two pathways including tyrosine metabolism and phenylalanine, tyrosine and tryptophan biosynthesis were the critical disturbed pathways associated with CAD severity in KTRs (Fig. 4B). Detailed and hypothetical metabolic pathway maps were generated based on the differential metabolites (Fig. 4C-D).

### Correlation analyses of differential metabolites and clinical data

**Fig. 5:**
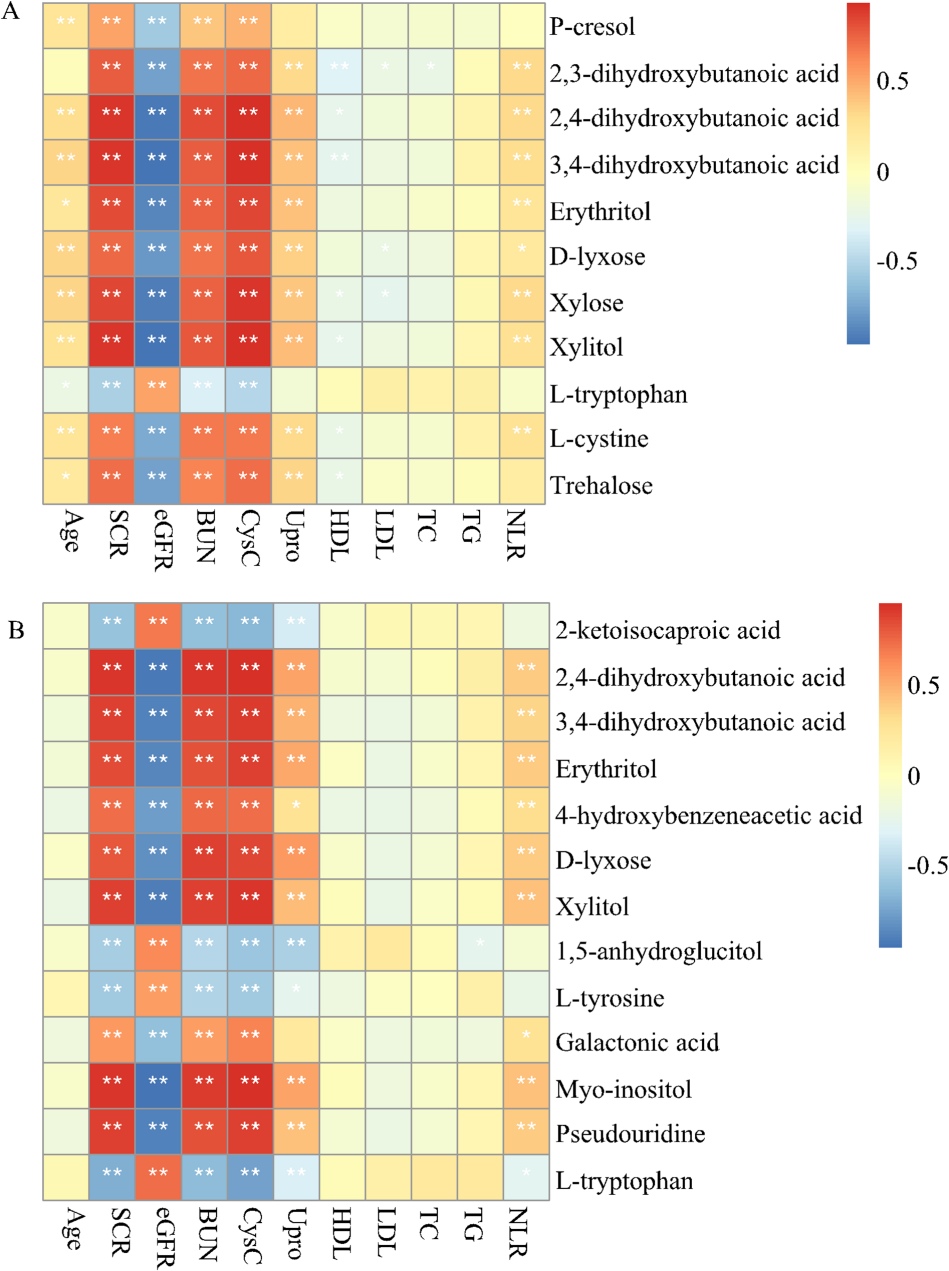
Correlation analysis of the selected metabolites and clinical data. (**A**) Spearman correlations of 11 differential metabolites and 11 clinical indicators in native CKD and HC patients. (**B**) Spearman correlations of 13 differential metabolites and 11 clinical indicators in KTRs. Note: *P < 0.05, **P < 0.01. SCR, serum creatinine; eGFR, estimated glomerular filtration rate; BUN, urea nitrogen; CysC, cystatin C; Upro, urine protein; HDL, high density lipoprotein; LDL, low density lipoprotein; TC, total cholesterol; TG, triglyceride; NLR, neutrophil-to-lymphocyte ratio

Spearman correlation analyses were conducted to investigate the correlations between differential metabolites and clinical parameters. In HC and native CKD cohorts, except for L-tryptophan, which showed a negative correlation with the kidney function-associated markers (SCR, BUN, CysC), the other 10 metabolites were positively related to those markers in CKD and HC cohorts (Fig. 5A). In the KTR cohort, 2-ketoisocaproic acid, 1,5-anhydroglucitol, L-tyrosine and L-tryptophan were negatively correlated with kidney allograft function, whereas the other 9 metabolites were positively correlated (Fig. 5B). In addition, the majority of the differential metabolites were positively correlated with the inflammatory indicator NLR in both CKD and KTR patients.

### Identification of differential metabolites between CKD and KTRs who were at the same kidney dysfunction stages

**Fig. 6:**
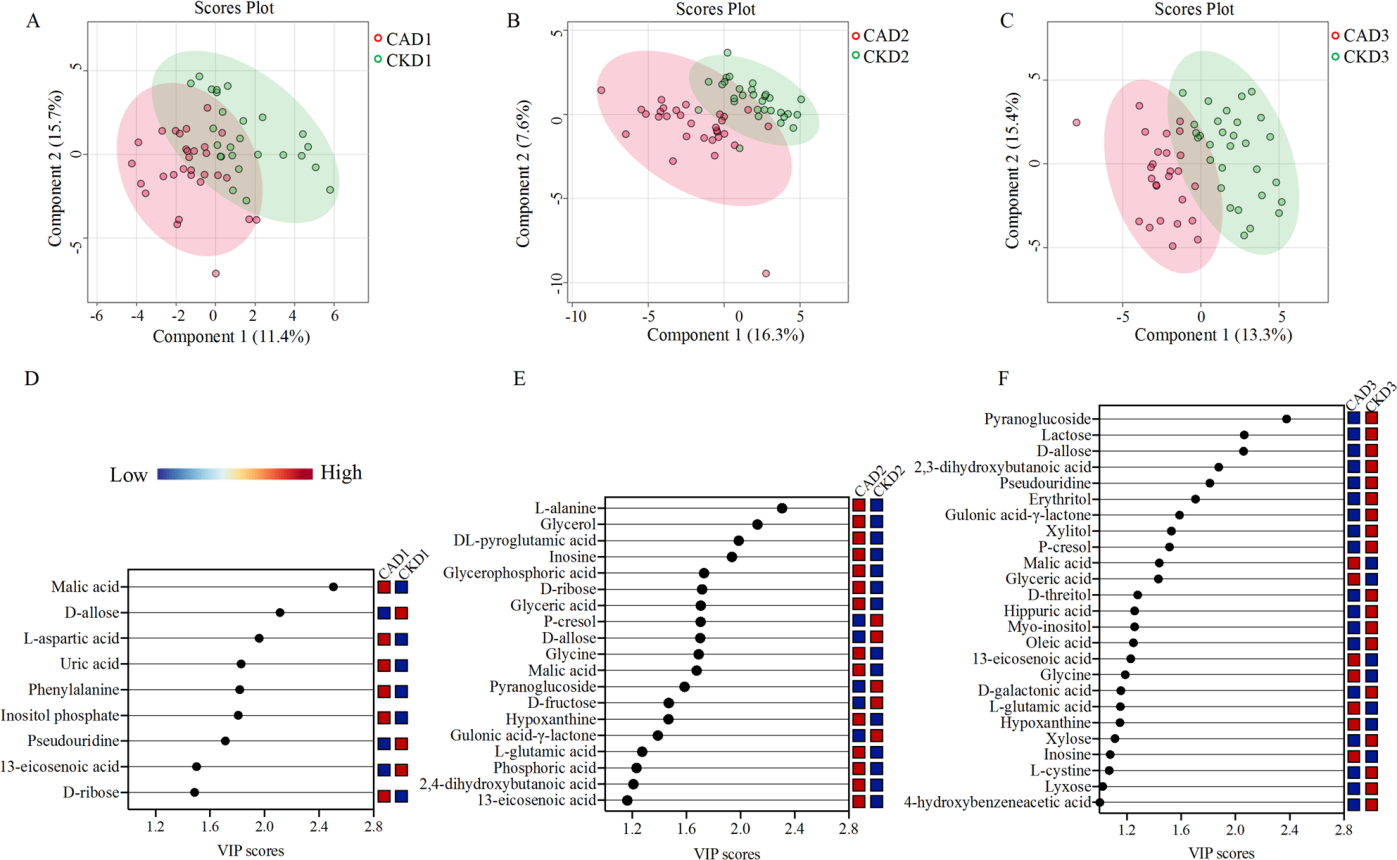
Identification of differential metabolites between CKD and KTRs who are at the same kidney dysfunction stages. **A**-**C**. PLS-DA score plots of serum metabolic profiles between CAD1 and CKD1 (**A**), CAD2 and CKD2 (**B**), and CAD3 and CKD3 (**C**). **D**-**F**. VIP score plots of the differential metabolites between CAD1 and CKD1 (**D**), CAD2 and CKD2 (**E**), and CAD3 and CKD3 (**F**) based on VIP > 1, FC > 1.2 or < 0.83, *P* < 0.05. Note: CKD, chronic kidney disease; KTR, kidney transplant recipient; PLS-DA, partial least squares-discriminant analysis; VIP, variable influence on projection; FC, fold change

Considering that lifelong immunosuppressive drugs and only one functional allogeneic kidney in KTRs may result in different metabolic alterations when compared to native CKD patients even if their renal functions are at the same stages, we compared the differential metabolites between native CKD and KTRs who were at the same kidney dysfunction stages. The baseline characteristics are presented in Table 3. In general, KTRs are younger and showed higher levels of cholesterol than those in CKD patients when their renal functions were at stage 2 or 3. KTRs in the CAD3 group showed significantly better renal function than those in the CKD3 group. PLS-DA multivariate analysis demonstrated that CKD patients were apparently separated from KTRs with similar eGFR levels (Fig. 6A and C). The metabolites responsible for the overall discrimination ability were also identified according to the pre-established criteria (VIP > 1, FC > 1.2 or < 0.83, and *P* < 0.05), which are commonly adopted in metabolomics studies to ensure both statistical and biological relevance [10, 11]. Interestingly, the worse the renal function, the more differential metabolites were identified between the CKD and CAD subgroups. 9, 19, and 25 differential metabolites were determined by comparing CKD1 and CAD1, CKD2 and CAD2, and CKD3 and CAD3, respectively (Fig. 6D and F). After intersection analysis of these differential metabolites, malic acid and D-allose were identified as common differential metabolites across the 3 subgroup comparative analyses. Although 13-eicosenoic acid was also selected as the common differential metabolite, its level showed inconsistent trends in inter-group comparisons.

**Table 3:** Comparison of characteristics between native CKD and KTR subgroups at the same kidney dysfunction stages

	Stage 1		Stage 2		Stage 3	
	CKD1	CAD1	CKD2	CAD2	CKD3	CAD3
Number	27	30	30	30	30	26
Age, years	40.8 ± 10.1	42.1 ± 11.2	47.4 ± 13.2	39.1 ± 8.6**	47.5 ± 15.5	37.4 ± 10.0**
Male, n (%)	16 (59.3%)	22 (73.3%)	21 (70.0%)	22 (73.3%)	22 (73.3%)	17 (65.4%)
**Kidney function**						
SCR (µmol/l)	97.5 ± 14.0	99.2 ± 17.5	156.3 ± 29.5	170.3 ± 26.0	561.2 ± 304.3	292.9 ± 82.5****
eGFR (ml/min/1.73m^2^)	75.2 ± 9.1	76.4 ± 10.0	42.5 ± 7.6	40.7 ± 6.8	11.9 ± 6.5	21.7 ± 4.8****
Serum urea, mmol/L	6.4 ± 2.3	6.1 ± 1.5	8.4 ± 2.7	10.2 ± 3.2*	20.9 ± 9.1	17.9 ± 7.1
Serum cystatin C, mg/L	1.3 ± 0.4	1.2 ± 0.2	1.9 ± 0.4	2.1 ± 0.6	4.6 ± 1.9	3.5 ± 1.0**
Urine protein, n						
0	12	17	5	13	2	4
+/-	5	6	4	4	1	4
+	3	5	4	6	4	5
++	6	2	10	4	11	11
+++	0	0	4	3	4	2
Unavailable	1	0	3	0	8	0
**Liver function**						
ALT, IU/L	17 (11–23)	13 (10–21)	17 (12–21)	13 (10–16)*	15 (8–27)	14 (10–22)
AST, IU/L	21 (15–25)	17 (15–21)	20 (16–24)	16 (14–19)*	14 (11–19)	19 (12–23)
**Lipid profile**						
Total cholesterol, mmol/L	4.20 (3.71–4.84)	5.03 (4.48–5.50)**	4.19 (3.43–5.05)	5.25 (4.51–5.77)**	4.21 (3.39–4.71)	4.96 (3.52–5.39)
Triglyceride, mmol/L	1.52 (1.25–1.65)	1.33 (0.98–2.04)	1.48 (1.15–2.28)	1.87 (1.35–2.42)	1.53 (1.14–2.07)	1.52 (1.05–2.27)
HDL-C, mmol/L	1.12 (1.03–1.36)	1.36 (1.08–1.53)*	1.07 (0.94–1.32)	1.28 (1.10–1.63)*	0.78 (1.00-1.41)	1.14 (1.00-1.73)*
LDL-C, mmol/L	2.68 (1.90–3.01)	3.02 (2.33–3.38)	2.56 (1.98–3.22)	2.97 (2.31–3.60)	2.15 (1.93–2.73)	2.60 (1.80–3.21)
**Others**						
NLR	1.93 (1.59–2.99)	2.11 (1.88–2.53)	2.60 (2.06–3.28)	2.47 (1.72–3.74)	2.71 (1.88–3.44)	4.25 (2.68–5.66)*

Abbreviations: CKD, chronic kidney disease; KTRs, kidney transplant recipients; CAD, chronic allograft dysfunction; SCR, serum creatinine; eGFR, estimated glomerular filtration rate; ALT, alanine aminotransferase; AST, aspartate transaminase; HDL-C, high-density lipoprotein cholesterol; LDL-C, low-density lipoprotein cholesterol; NLR, neutrophil-to-lymphocyte ratio

Note: * P < 0.05; **P < 0.01; ***P < 0.001; ****P < 0.0001

## Discussion

Metabolic disorders are highly prevalent among various kidney diseases. In theory, loss of kidney function is associated with certain enzymatic activity and metabolite clearance capacity; therefore, serum metabolites show the potential to be linked to the changes in kidney function. In this study, we used untargeted metabolic GC-MS analysis to vertically compare the metabolite alterations in patients with different renal functions in separate cohorts of native CKD and transplanted CKD patients. We identified 11 and 13 metabolites as the common differential metabolites associated with different levels of kidney function in the native CKD and KTR cohorts, respectively. Furthermore, we identified that L-tryptophan, D-lyxose, xylitol, erythritol, 3,4-dihydroxybutanoic acid and 2,4-dihydroxybutanoic acid were closely related to kidney function in both types of CKD. Pathway analysis revealed that pentose and glucuronate interconversions was significantly enriched among the native CKD subgroups, while the tyrosine metabolism pathway and phenylalanine, tyrosine and tryptophan biosynthesis pathways were enriched in KTR subgroups. A horizontal comparison of native CKD patients and KTRs at the same stages demonstrated that malic acid and D-allose are the common differential metabolites, which may be influenced by immunosuppressive drugs and allogeneic immune regulation.

We acknowledge that a substantial portion of our results, such as the association of tryptophan/kynurenine pathway dysregulation and elevated polyols with kidney dysfunction, serves to validate and extend prior observations [12–14]. The primary novel contribution of our study lies in the direct cross‑cohort comparison between non‑transplant CKD patients and KTRs at equivalent stages of kidney dysfunction. To select specific metabolites that were closely associated with cross-sectional differences in kidney function in both native and transplanted CKD settings, we utilized the intersection strategy and 6 metabolites were finally identified. It has been well-established that the tryptophan-kynurenine pathway plays an important role in the occurrence and progression of acute and chronic kidney diseases [13]. Consistently, we found that L-tryptophan—one of the 6 identified differential metabolites—decreased dramatically with more severe kidney dysfunction. In contrast, kynurenine, the first product of L-tryptophan metabolism, was not filtered out based on our selection criteria. Tryptophan is one of the nine essential amino acids that can be obtained only from daily protein intake [15]. Although dietary protein restriction in CKD patients could affect circulating tryptophan levels, our data support that the observed tryptophan decrease is not solely a dietary influence. The participants provided fasting serum samples, minimizing acute dietary confounding. In addition, tryptophan can be broken down into kynurenine by the rate-limiting enzyme, indoleamine 2,3-dioxygenase (IDO) catabolism [16]. It is widely accepted that the circulating kynurenine/ L-tryptophan ratio is a valuable clinical benchmark for IDO-1 activation and reflects the state of immune activation or tolerance. Our data demonstrated that the ratio of kynurenine/ L-tryptophan significantly increased in patients with worse kidney function, suggesting that the IDO activity might be enhanced in those with advanced CKD or CAD (Supplementary Fig. 1). Furthermore, advanced CKD or CAD patients usually have a hyperinflammatory status, which is the most potent inducer of IDO activity [17–20], confirming our observations and hypothesis in this study. In addition, these data were consistent with those of a previously published study that showed that the kynurenine/ L-tryptophan ratio was correlated with annual changes in eGFR and increased CKD incidence [21], robustly supporting the reliability of our metabolomic data.

In addition to amino acids, we also found that several sugars and sugar alcohols (xylitol, D-lyxose, and erythritol) were significantly elevated with kidney disease severity. Our results are consistent with a previous study conducted by Kassaporn Duangkumpha et al., who identified elevated levels of D-xylose, D-maltose, meso-erythritol, and myo-inositol in patients with diabetic kidney failure compared to those with diabetes alone [22]. Xylitol is a naturally occurring intestinal metabolite. Beyond its us as a sugar substitute, it has been shown to inhibit lipopolysaccharide-induced inflammatory cytokines and angiogenic activity [23, 24]. In this study, we found that serum xylitol increased significantly with more severe kidney dysfunction in both non-transplant CKD patients and KTRs. The mechanism underlying this elevation is likely multifactorial. Vanlede et al. reported that urinary excretion of xylitol was significantly higher in children with CKD stage 3–5 compared to those with stage 1–2, suggesting that increased urinary loss does not account for the elevated serum levels observed in advanced CKD [25]. Moreover, an early study by Spitz et al. in uremic patients demonstrated that xylitol is rapidly cleared from the blood and that urinary excretion accounts for less than 1% of the administered dose [26]. This indicates that renal excretion alone is unlikely to be the primary driver of elevated serum xylitol in advanced kidney disease, because even in uremia the clearance capacity for exogenous xylitol remains efficient. An alternative explanation is that enhanced endogenous synthesis, possibly through altered gut–kidney axis metabolism [27], may contribute to the accumulation of circulating xylitol. Indeed, Tan et al. demonstrated that fecal xylitol levels were significantly lower in CKD patients and in a unilateral ureteral obstruction mouse model, and xylitol supplementation exerted protective effects against renal fibrosis in vivo [27]. However, the relationship between gut-derived xylitol and circulating xylitol levels remains unclear, and whether elevated serum xylitol represents a compensatory protective response warrants further investigation. Erythritol, another sugar alcohol, was also elevated in patients with advanced CKD and CAD. Erythritol is a 4-carbon polyol that occurs naturally in fruits and fermented foods and can also be endogenously synthesized from glucose through the pentose phosphate pathway (PPP) [28]. The mechanisms underlying elevated serum erythritol in CKD remain debated. Given that more than 90% of absorbed erythritol is excreted unchanged in the urine [29], the impaired renal clearance undoubtedly contributes to its accumulation in advanced stages. Moreover, vanlede et al. observed that urinary excretion of several polyols was already elevated in early-stage CKD compared to healthy controls [25]. This finding suggests that increased endogenous synthesis may occur in early disease stages, whereas reduced clearance becomes more dominant in advanced stages. Therefore, the elevated serum erythritol observed in our patients likely reflects a combination of enhanced synthesis in early CKD and reduced excretion in later stages. Although the relative contributions of endogenous synthesis and urinary excretion fractions remains unclear, elevated serum erythritol may serve as a potential biomarker of kidney disease. This is support by Haukka et al., who found that erythritol, in combination with two other metabolites, was the best set of variables for predicting the development of microalbuminuria after metabolomic selections, and they may improve nephropathy screening [30]. Interestingly, 2,4-dihydroxybutanoic acid and 3,4-dihydroxybutanoic acid were significantly elevated; however, limited data have explored the association of these two substances with kidney diseases. In cross-sectional analyses, 2,4-dihydroxybutanoic acid and 3,4-dihydroxybutanoic acid were positively associated with microalbuminuria and macroalbuminuria versus normoalbuminuria and inversely associated with eGFR [14]. These findings are consistent with the findings observed in the present study. However, the mechanisms underlying the elevation of these metabolites and their functional roles in kidney disease require further investigation.

Based on the identified differential metabolites in separate cohorts, pathway enrichment analyses revealed that the pentose and glucuronate interconversions pathway was significantly affected in the native CKD cohort, whereas phenylalanine, tyrosine and tryptophan biosynthesis pathway and tyrosine metabolism pathway were the most affected pathways in transplanted CKD patients. It has been established that immunosuppressants such as calcineurin inhibitors and antimetabolites are known to directly alter metabolic pathways [31, 32]. Therefore, we cannot definitely conclude whether the metabolic signatures specific to KTRs are driven primarily by allograft pathology, the pharmacological effects of immunosuppression, or most plausibly, an interaction of both. Although KT can improve kidney function in patients with ESKD, it does not completely resolve the complications such as bone disease and metabolic disorders [33]. Our data also supported that even though native CKD patients and KTRs were at the same disease stages, they were obviously separated in PLS-DA analytic plots based on metabolite distribution. In subgroups with more severe kidney dysfunction, a greater number of differential metabolites were identified between the native CKD and KTRs subgroups. This may be related to the much more complicated internal environment in patients with advanced kidney diseases. Additionally, malic acid and D-allose were the two differential metabolites that showed the same alteration trends across the 3 comparison groups, with KTRs showing significantly higher levels of malic acid, but much lower levels of D-allose than those in native CKD patients. However, these two metabolites did not correlate with kidney dysfunction in our study. Whether these metabolites are involved in the pathological process of kidney diseases remains to be elucidated.

This study has some limitations. First, the relatively modest sample size within each subgroup may have limited the statistical power to detect metabolites with smaller effect sizes, potentially leading to an underestimation of the true number of metabolites associated with kidney function. Second, because of the limitations of the GC-MS method in metabolomics (imprecise quantification), precise quantification of the identified differential metabolites, such as using high-performance liquid chromatography-MS/MS (HPLC-MS/MS), should be conducted to verify our preliminary findings. Third, the lack of age-matched HCs in this study may partially contribute to the observed metabolic differences, and thus the results should be interpreted with caution when compared with HCs. Fourth, due to insufficient renal biopsy data, the original causes of native CKD were lacking in this study. Therefore, we could not conduct further subgroup analyses to explore the associations between metabolites and disease pathological characteristics.

## Conclusions

Using an untargeted GC-MS platform, we identified that the downregulation of L-tryptophan, and the upregulation of D-lyxose, xylitol, erythritol, 3,4-dihydroxybutanoic acid, and 2,4-dihydroxybutanoic acid were closely correlated with kidney function, regardless of CKD patients receiving kidney transplantation, suggesting that the panel of 6 common differential metabolites may be the potential markers for kidney disease severity. However, due to the cross-sectional design, longitudinal studies are needed to evaluate whether these metabolites can predict future kidney function decline. We also identified 5 and 7 metabolites that were specifically associated with CKD severity in non-transplant patients and transplant patients, respectively. Pathway enrichment analyses revealed that pentose and glucuronate interconversions was impaired in patients with advanced native CKD, and tyrosine metabolism was enhanced in KTRs with advanced CAD. However, we could not determine whether the alterations of these metabolites or metabolic pathways is the cause or consequence of kidney dysfunction. In the future studies, this issue should be addressed, which may provide clues for further exploration of the therapeutic targets and promising biomarkers for kidney diseases. Notably, KTRs are a unique population of patients with CKD, whose metabolism might be significantly affected by the lifelong use of immunosuppressive medications and transplant-related complications, such as infections and rejections. These factors should be taken into consideration when investigating the metabolic alterations in KTRs.

## Supplementary Information

Below is the link to the electronic supplementary material.

Supplementary Material 1

## Data Availability

The raw metabolomics data generated in this study have been deposited in the National Genomics Data Center (NGDC) OMIX repository under accession code OMIX013657.

## Abbreviations

GC-MS: Gas chromatography-mass spectrometry
CKD: Chronic kidney disease
ESKD: End-stage kidney disease
KT: Kidney transplantation
eGFR: Estimated glomerular filtration rate
KTR: Kidney transplant recipients
CAD: Chronic allograft dysfunction
SCR: Serum creatinine
HC: Healthy control
QC: Quality control
RI: Retention index
RSD: Relative standard deviation
VIP: Variable influence projection
FC: Fold changes
PLS-DA: Partial least squares-discriminant analysis
NLR: Neutrophil-to-lymphocyte ratio
IDO: Indoleamine 2,3-dioxygenase
ABMR: Antibody-mediated rejection
