# Supplementary material for: Serum metabolites in relation to kidney function in non-transplant and kidney transplant settings using gas chromatography-mass spectrometry
Source: BMC Nephrol. 2026 Apr 29;27:373. doi: 10.1186/s12882-026-05000-1 (PMC13277178; doi:10.1186/s12882-026-05000-1)
Supplement: Supplementary file 1 — Supplementary Material 1 [file 12882_2026_5000_MOESM1_ESM.docx]

**Supplementary materials**

**Supplementary Figure 1** Quantification of L-tryptophan, Kynurenine, and Kynurenine/L-tryptophan ratio among native CKD and KTR subgroups. A. Relative intensity of L-tryptophan among CKD subgroups and HC group; B. Relative intensity of Kynurenine among CKD subgroups and HC group; C. Calculation of Kynurenine/L-tryptophan ratio among CKD subgroups and HC group; D. Relative intensity of L-tryptophan among KTR subgroups; E. Relative intensity of Kynurenine among KTR subgroups; F. Calculation of Kynurenine/L-tryptophan ratio among KTR subgroups.

Note: ^#^Compared with HC group, ^#^ *P*<0.05; ^##^*P*<0.01; ^###^*P*<0.001; ^####^*P*<0.0001; **P*<0.05; ***P*<0.01; ****P*<0.001; *****P*<0.0001. CKD, chronic kidney disease; KTR, kidney transplant recipient; CAD, chronic allograft dysfunction; HC, healthy control.
